# Characteristics of gut microbiota of premature infants in the early postnatal period and their relationship with intraventricular hemorrhage

**DOI:** 10.1186/s12866-024-03675-w

**Published:** 2024-12-02

**Authors:** Yunlong Zhao, Shan Li, Rui Zhang, Xin Zhang, Qiuyue Shen, Xingyun Zhang, Tian Tian, Xinlin Hou

**Affiliations:** 1 Department of Pediatrics, Peking University First Hospital, Beijing, China; 2 Academy for Advanced Interdisciplinary Studies, Peking University, Beijing, China

**Keywords:** Gut microbiota, Intraventricular hemorrhage, Premature infant

## Abstract

**Background:**

Studies have shown correlations between gut microbiota and neurocognitive function, but little was known about the early postnatal gut microbiota and intraventricular hemorrhage (IVH). We aimed to explore the characteristics of gut microbiota in premature infants and their relationship with IVH, further exploring potential therapeutic targets.

**Methods:**

Premature infants delivered at Peking University First Hospital from February 2023 to August 2023 were recruited as a cohort. Feces samples were collected on postnatal days 1, 3, and 5. Premature infants were divided into normal, mild IVH, and severe IVH groups based on cranial ultrasound. 16S rRNA amplicon sequencing technology was used to determine the fecal microbiota, and the results were analyzed.

**Results:**

Thirty-eight premature infants were enrolled. There was a significant difference in alpha and beta diversity among the three groups. The relative abundance of *E. coli* and *A. muciniphila* was different among the three groups. Further random forest analysis indicated that *S. lutetiensis*, *L. mirabilis*, and *N. macacae* can effectively distinguish premature infants with IVH. Finally, the phylogenetic investigation of communities by reconstruction of unobserved states2 (PICRUSt2) functional gene analysis predicted significant differences in energy metabolism, carbohydrate metabolism, metabolism of cofactors and vitamins, and membrane transport between normal and severe IVH groups.

**Conclusions:**

The gut microbiota in the early postnatal period of premature infants is closely associated with the IVH status. As age increases, the differences in gut microbiota of premature infants with different degrees of IVH continue to increase, and the trend of changes with severity of IVH becomes more and more obvious. *E. coli*, *A. muciniphila*,* S. lutetiensis*,* L. mirabilis*,* N. macacae*,* G. haemolysans*,* and S. oralis* can effectively distinguish between IVH infants and normal premature infants. The results indicate that gut microbiota is expected to provide effective therapeutic targets for the diagnosis and treatment of IVH.

**Supplementary Information:**

The online version contains supplementary material available at 10.1186/s12866-024-03675-w.

## Background

With the improvement of neonatal critical care, the mortality of premature infants decreased markedly. However, there was no significant reduction in severe intraventricular hemorrhage (IVH) [1, 2]. IVH with an incidence of approximately 23% [3–5], is the most common complication of extremely premature infants [6]. The mortality rate of infants with severe IVH (grades III and IV) is approximately 10%, and could reach to 67.6–88.7% withholding and withdrawing of life-sustaining treatment in some report [7]. The cognitive or motor disorders occurring in 35 -50% of survivors. Approximately 22% of infants with mild IVH (grades I and II) will experience neurological sequelae [8]. Lacking effective prediction and intervention parameters, when infants show obvious symptoms such as seizures, they often develop into severe IVH [9].

The well-known “gut-brain axis” theory emphasizing the interaction between gut microbiota and the nervous system, has been proved in autism spectrum disorder, attention deficit hyperactivity disorder (ADHD), and many other diseases [10–13]. This bidirectional regulation is crucial for maintaining human health [14]. Studies have shown that gut microbiota may affect neurocognitive function and play a regulatory role [15–18]. After depleting the gut microbiota using antibiotic treatment from weaning onwards, mice showed significant cognitive impairment, altered dynamics of the tryptophan metabolic pathway, and significantly reduced brain-derived neurotrophic factor (BDNF), oxytocin and vasopressin expression in the adult brain [16]. Mice infected with pathogenic bacteria significantly improved their impaired memory ability after supplementing with probiotics [17]. Children with autism or ADHD often have gastrointestinal dysfunction, which can be improved by supplementing probiotics [11, 12].

Studies on infants have shown a correlation between gut microbiota and neurocognitive function. Higher alpha diversity of gut microbiota in small for gestational age (GA) infants on postnatal day 3 was found to be associated with poor communication performance at 6 months of age [19]. Another study found an association between the gut microbiota of infants aged 3–6 months and communication and fine motor skills at 3 years old [20]. The alpha diversity of gut microbiota in 1-year-old infants can predict cognitive function at 2 years old [21].

The neonatal period is critical for the colonization and development of gut microbiota, which plays an important role in promoting neural function development [21–23]. However, it is still unknown whether the gut microbiota of premature infants is involved in the occurrence of IVH, and the relationship between gut microbiota and IVH.

In this study, we aim to explore the characteristics of gut microbiota of IVH infants in the early postnatal period by high-throughput sequencing technology. We try to explore potential microbiota biomarkers targeting early treatment and diagnosis of post-IVH cognitive impairment in premature infants, thus improving the prognosis of premature infants with IVH.

## Materials and methods

### Study protocol

This study adopted a cohort design, targeting premature infants with GA < 37 weeks born at Peking University First Hospital from February to August 2023. Once enrolled, infants’ feces were collected on postnatal days 1, 3, and 5 (Day 1, Day 3, Day 5), and the fecal microbiota was determined using 16S rRNA amplicon sequencing technology. According to the cranial ultrasound result at 3 days, 1 week, and 2 weeks after birth, the enrolled infants were divided into three groups: normal, mild IVH, and severe IVH group. The severity of IVH was divided into grades I-IV using the Papile grading method, with grades I-II being mild and grades III-IV being severe. Infants were followed up regularly thereafter until corrected full-term age.

### Subjects

#### Inclusion criteria

(a) Informed consent of the legal guardian; (b) Premature infants (GA < 37 weeks); (c) Perform cranial ultrasound examination within 1 week after birth.

#### Exclusion criteria

(a) Abandoning treatment due to social factors; (b) Brain injury other than IVH during the neonatal period; (c) diagnosed with other neurological diseases.

### Methods

#### Data and sample collection

Clinical data, including gender, GA, birth weight, severity of IVH, mode of delivery, Apgar score, antenatal corticosteroid use, prenatal antibiotic exposure, early postnatal antibiotic use, early feeding methods, and complications, were collected. Fecal samples were collected on sterilized diapers by well trained nurses on Day 1, Day 3, and Day 5. Fecal samples were temporarily in an ice box and stored in sterile cryotubes at -80℃ within half an hour then transferred to laboratory using dry ice within 1 month of sample collection [24]. Samples collected from blank diapers were used for sequencing control to assess contamination.

#### DNA extraction and gut microbiota sequencing

This study used 16S rRNA amplicon sequencing technology to determine the fecal microbiota. The genomic DNA from fecal samples was extracted utilizing the TGuide S96 Magnetic Soil/Stool DNA Kit (Tiangen Biotech (Beijing) Co., Ltd.), following the manufacturer’s protocol. The quality and quantity of the extracted DNA were assessed by gel electrophoresis on a 1.8% agarose gel, and the DNA concentration and purity were measured using a NanoDrop 2000 UV-Vis spectrophotometer (Thermo Scientific, Wilmington, USA).

The full-length 16S rRNA gene was amplified with primer pairs 27 F: AGRGTTTGATYNTGGCTCAG and 1492R: TASGGHTACCTTGTTASGACTT. Both the forward and reverse 16S primers were tailed with sample-specific PacBio barcode sequences to allow for multiplexed sequencing. The KOD One PCR Master Mix (TOYOBOLife Science) was used to perform 25 cycles of polymerase chain reaction (PCR) amplification, with initial denaturation at 95 °C for 2 min, followed by 25 cycles of denaturation at 98 °C for 10 s, annealing at 55 °C for 30 s, and extension at 72 °C for 90 s, and a final step at 72 °C for 2 min. The total of PCR amplicons were purified with VAHTSTM DNA Clean Beads (Vazyme, Nanjing, China) and quantified using the Qubit dsDNA HS Assay Kit and Qubit 3.0 Fluorometer (Invitrogen, Thermo Fisher Scientific, Oregon, USA). After the individual quantification step, amplicons were pooled in equal amounts. SMRTbell libraries were prepared from the amplified DNA by SMRTbell Express Template Prep Kit 2.0 according to the manufacturer’s instructions (Pacific Biosciences). Purified SMRTbell libraries from the pooled and barcoded samples were sequenced on a PacBio Sequel II platform (Beijing Biomarker Technologies Co., Ltd., Beijing, China) using Sequel II binding kit 2.0.

#### Bioinformatics analysis

Using the SMRT Link software (version 8.0) to filter and demultiplex the raw reads generated from sequencing to obtain the circular consensus sequencing (CCS) sequences. And then using the lima (version 1.7.0) to assign the CCS sequences to the corresponding samples based on their barcodes. CCS sequences containing no primers and those reads beyond the length range (1200–1650 bp) were discarded through the recognition of forward and reverse primers and quality filtering using the Cutadapt [25] (version 2.7) quality control process. The UCHIME algorithm [26] (version 8.1) was employed in detecting and removing chimera sequences to obtain the effective CCS, which were clustered at a similarity level of 97.0% to obtain operational taxonomic units (OTU) by USEARCH [27] (version 10.0), and the OTUs counts less than 2 in all samples were filtered. The OTUs sequences were compared with the SILVA database [28] (release 138.1) based on the Naive Bayes classifier in QIIME2 [29] with a confidence threshold of 70%, in order to obtain the corresponding species’ taxonomic information. Bioinformatics analysis was conducted on the gut microbiota in all collected samples while GA and birth weight (BW) were set as environmental factors.

Alpha diversity analysis: Using QIIME2 [29] and R software to calculate and display the alpha diversity respectively. The Chao1 index measuring species richness, and the Shannon index measuring species diversity [30] of each sample were calculated. Rarefaction curves based on Shannon index were drawn to determine whether the sample size was reasonable.

Beta diversity analysis: Using QIIME to calculate the beta diversity. Principal co-ordinates analysis (PCoA) and analysis of similarities (ANOSIM) were conducted to compare the differences in species diversity, structure, and community composition between different groups [31].

Analysis of inter-group significant differences: Linear discriminant analysis effect size (LEfSe) [32] was used to obtain differential dominant microbiota in different samples. Analysis of variance (ANOVA) was used to obtain relative abundance differences. Using the randomForest function (version 4.6–10) in R software (version 3.1.1) for random forest analysis [33], to obtain key species that can distinguish different groups of samples through the MeanDecreaseGini index [34].

Association analysis: Redundancy analysis / canonical correspondence analysis (RDA/CCA analysis) was used to reflect the relationship between microbial communities or samples and environmental factors by the package vegan in R software [35].

Functional gene prediction analysis: The composition and differences of the microbial genome database Kyoto encyclopedia of genes and genomes (KEGG) metabolic pathways were analyzed in the phylogenetic investigation of communities by reconstruction of unobserved states2 (PICRUSt2) [36], to observe the differences and changes in functional genes of microbial communities in metabolic pathways among different groups of samples.

#### Statistical analysis

Clinical data were analyzed by IBM SPSS Statistics (Version 26.0), and graphs were drawn using GraphPad Prism (Version 8.0). Measurement data conformed to a normal distribution were expressed as mean ± standard deviation (x ± s). For comparison between two groups, Student’s t-test was used, otherwise using ANOVA analysis. Measurement data not conformed to a normal distribution were represented by the median (P25, P75). For comparison between two groups, the Mann Whitney U test was used, otherwise using the Kruskal Wallis H test. Comparison between enumeration data groups was performed using χ 2 test. For the correlation analysis of two quantitative datasets, Pearson correlation analysis was used if they conform to a bivariate normal distribution, otherwise using Spearman correlation analysis. *P* < 0.05 indicated a statistically significant difference.

## Results

### Clinical information

**Table 1:** Clinical characteristics of the normal, mild and severe groups

Descriptive variable		Normal group *n* = 21	Mild group *n* = 8	Severe group *n* = 9	*P* value
General information					
	Male, n (%)^a^	6(28.6)	6(75)	7(77.8)	0.011
	Birth weight, g, mean (SD)^b^	2115.5(592.9)	1290(346.6)	898.9(189.9)	< 0.001
	Gestational age, weeks, mean (SD)^b^	33.9(2.4)	28.9(1.8)	26.5(1.3)	< 0.001
Perinatal history					
	Infants from multiple pregnancy, n (%)^a^	10(47.6)	3(37.5)	2(22.2)	0.407
	Apgar Score 1 min, median (IQR)^c^	10(2)	8(3)	5(5)	0.08
	Apgar Score 5 min, median (IQR)^c^	10(1)	9(1)	9(3)	0.059
	Apgar Score 10 min, median (IQR)^c^	10(0)	10(0)	10(0)	0.496
	Initial pH, mean (SD)^b^	7.31(0.06)	7.35(0.17)	7.32(0.07)	0.667
	Vaginal delivery, n (%)^a^	7(33.3)	5(62.5)	6(66.7)	0.150
	Antenatal Corticosteroids, n (%)^a^	15(71.4)	6(75)	6(66.7)	0.930
	Fetal Distress, n (%)^a^	6(28.6)	0(0)	1(11.1)	0.086
	Maternal PROM, n (%)^a^	10(47.6)	6(75)	3(33.3)	0.206
	Prenatal Antibiotics, n (%)^a^	13(61.9)	7(87.5)	8(88.9)	0.167
	No. of IVAB during first week of life, day, median (IQR)^c^	5(1)	6(2)	7(0)	< 0.001
	UU infection, n (%)^a^	2(9.5)	1(12.5)	0(0)	0.592
Feeding Regime					
	DOL of first MEBM, day, median (IQR)^c^	3.0(1)	3(4)	7(9)	0.025
	DOL of TEN, day, median (IQR)^c^	7(4)	21(19)	37(24.3)	< 0.001
	Respiratory support requirement				
	No. dosage of PS administration, median (IQR)^c^	0(1)	1(1)	4(2)	< 0.001
	No. of d requiring mechanical ventilation, day, median (IQR)^c^	0(1)	1(3)	11.5(11)	< 0.001
	No. of d requiring noninvasive ventilation day, median (IQR)^c^	0(5)	6.5(8)	31(21)	< 0.001
	No. of d requiring NP oxygen, day, median (IQR)^c^	0(0)	15(37)	45(37)	< 0.001
Complications					
	NEC, n (%)^a^	1(4.8)	1(12.5)	2(22.2)	0.194
	hsPDA, n (%)^a^	1(4.8)	2(25)	4(44.4)	0.032
	ROP, n (%)^a^	1(4.8)	1(12.5)	3(33.3)	0.131
	BPD, n (%)^a^	3(14.3)	5(62.5)	9(100)	< 0.001

*IVH: intraventricular hemorrhage; PROM*,* premature rupture of fetal membrane; IVAB*,* intravenous antibiotics; UU*,* Ureaplasma urealyticum; DOL*,* day of life; MEBM*,* maternally expressed breast milk; TEN*,* total enteral nutrition; PS*,* pulmonary surfactant; NP*,* nasal prong; NEC*,* necrotizing enterocolitis; hsPDA*,* hemodynamic significant patent ductus arteriosus; ROP*,* retinopathy of prematurity*^;*a*^*Fisher’s exact test;*^*b*^*ANOVA test;*^*c*^*Kruskal-Wallis test.*

Thirty-eight premature infants were enrolled, including 21 in the normal group, 8 in the mild IVH group, and 9 in the severe IVH group (shown in Table 1). There were significant differences (*p* < 0.05) in day of life (DOL) of first maternally expressed breast milk (MEBM), total enteral feeding time, and respiratory support (number of pulmonary surfactants uses, invasive ventilator use, and oxygen uptake time), but no statistically significant difference in prenatal and delivery history among different groups. In terms of complications, there were significant differences in the hemodynamic significance of patent ductus arteriosus (PDA) and bronchopulmonary dysplasia among different groups, but no significant difference in the incidence of neonatal necrotizing enterocolitis and premature retinopathy.

One hundred one fecal samples were collected from 38 premature infants, including 33 samples on Day 1 (21 in the normal group, 5 in the mild group, and 7 in the severe group), 33 samples on Day 3 (20 in the normal group, 6 in the mild group, and 7 in the severe group), and 35 samples on Day 5 (20 in the normal group, 6 in the mild group, and 9 in the severe group). In total, there were 61 in the normal group, 17 in the mild IVH group, and 23 in the severe IVH group (shown in Supplementary Table 1).

### Gut microbiota analysis

In this study, each sample generated at least 8,457 CCS sequences, with an average of 12,859 CCS sequences and 12,658 effective CCS sequences after processing. 4,617 OTUs were obtained at the clustering similarity level of 97.0%. Samples collected from blank diapers were conducted as the negative control. The negative control group did not show any bands after DNA extraction and amplification, so 16s rRNA sequencing analysis was not performed.

#### Adequacy judgment of sample sequencing quantity

All Rarefaction curves based on Shannon index in this study tended to flatten out (shown in Supplementary Fig. 1), indicating the sample sequence was sufficient for data analysis.

#### Alpha diversity analysis

**Fig. 1:**
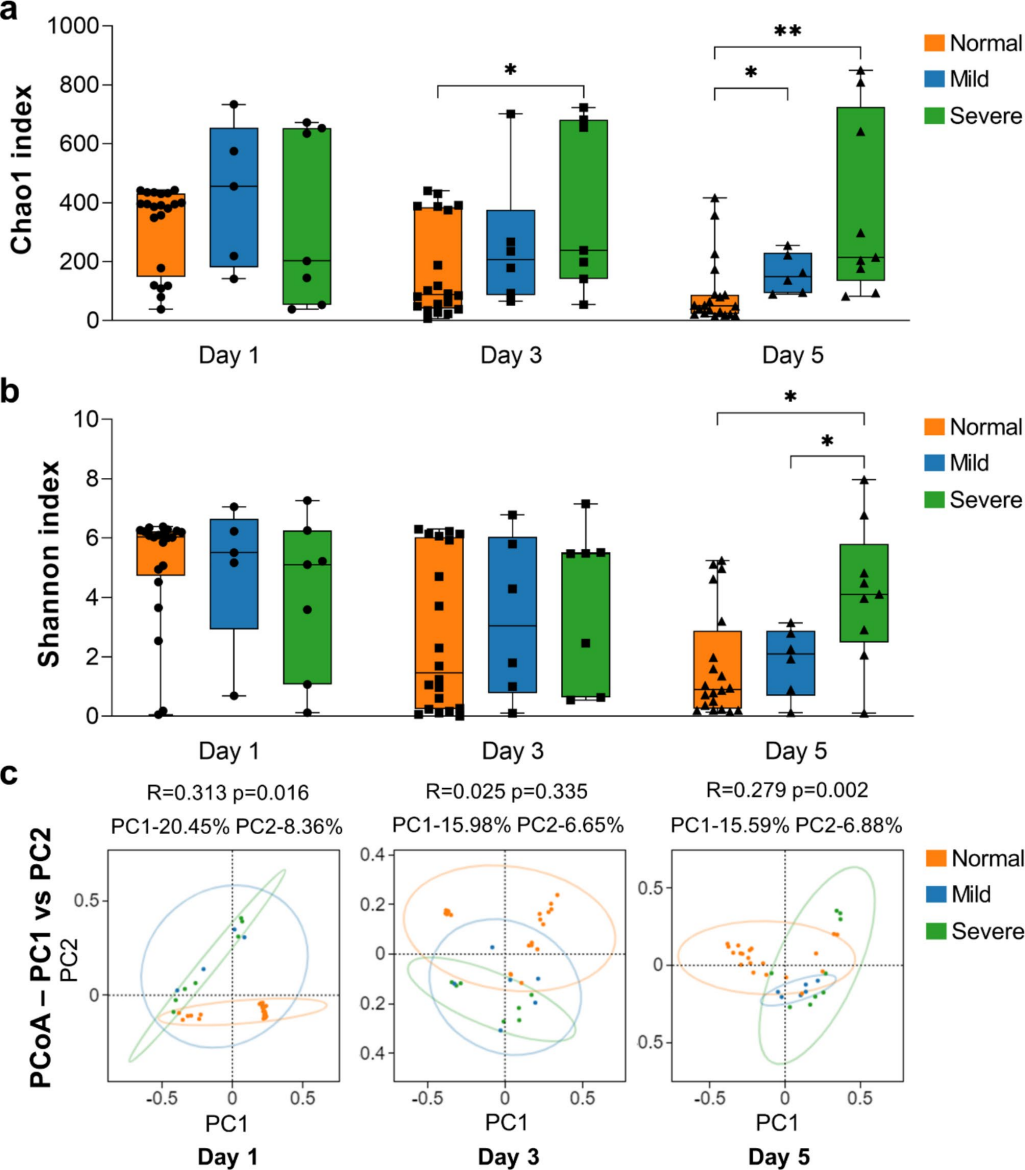
The alpha and beta diversity analysis between normal, mild, and severe groups. Differences in (**a**) Chao1 index measuring species richness in alpha diversity, (**b**) Shannon index measuring species diversity in alpha diversity, and (**c**) PCoA and ANOSIM analysis measuring beta diversity

As shown in Fig. 1. a-b, we found there was no significant difference in alpha diversity between the normal, mild IVH and severe IVH group on Day 1. When it comes to Day 3, alpha diversity between different groups began to differ. There was a significant difference in species richness between the normal and severe IVH group, but no significant difference in species diversity among the three groups. When the time reaches Day 5, the difference in alpha diversity between different groups becomes more significant. There was a significant difference in species richness between the normal and IVH group (mild and severe IVH group), and a significant difference in species diversity between the severe IVH group and the other two groups. In addition, we found that as age increased, the trend of alpha diversity changing with the severity of IVH became more and more obvious, that is, as the severity of IVH increased, alpha diversity continued to increase.

Interestingly, we found that the changes in alpha diversity with age varied among different IVH groups: in the severe group, the alpha diversity of gut microbiota did not change with age, while the normal group and mild IVH group showed a trend of decreasing diversity with age.

#### Beta diversity analysis

As shown in Fig. 1. c, through PCoA and ANOSIM analysis, we found differences in the beta diversity of gut microbiota in premature infants with different IVH severity levels on Day 1 (*R* = 0.313, *p* = 0.016), Day 3 (*R* = 0.025, *p* = 0.335), and Day 5 (*R* = 0.279, *p* = 0.002). These results indicate there were differences in the community composition and structure of gut microbiota in premature infants with different IVH severity levels. Further analysis of inter-group significant differences can be conducted.

#### Analysis of inter-group significant differences

**Fig. 2:**
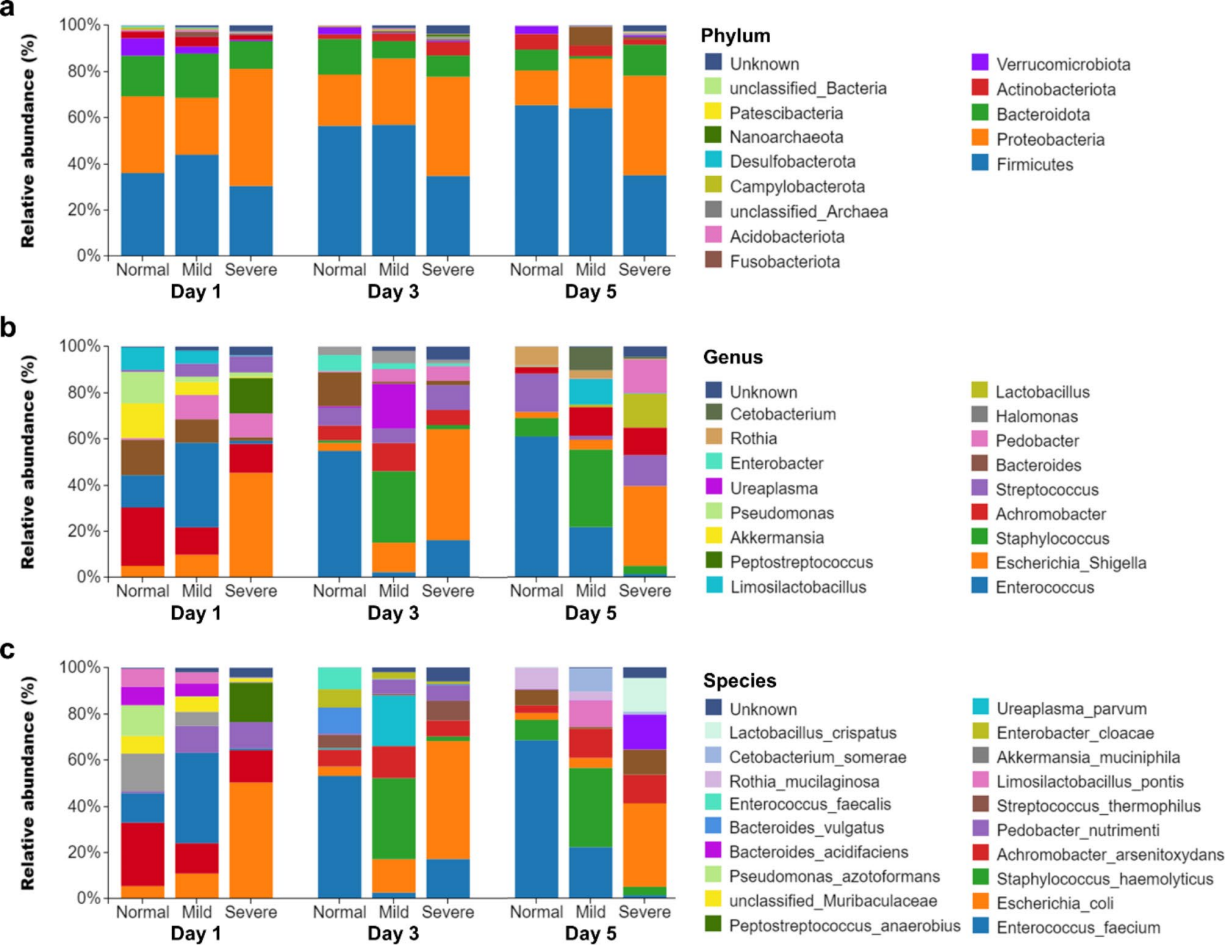
The analysis of species distribution differences between normal, mild, and severe groups. The species distribution bar charts at the (**a**) phylum, (**b**) genus, and (**c**) species level

**Fig. 3:**
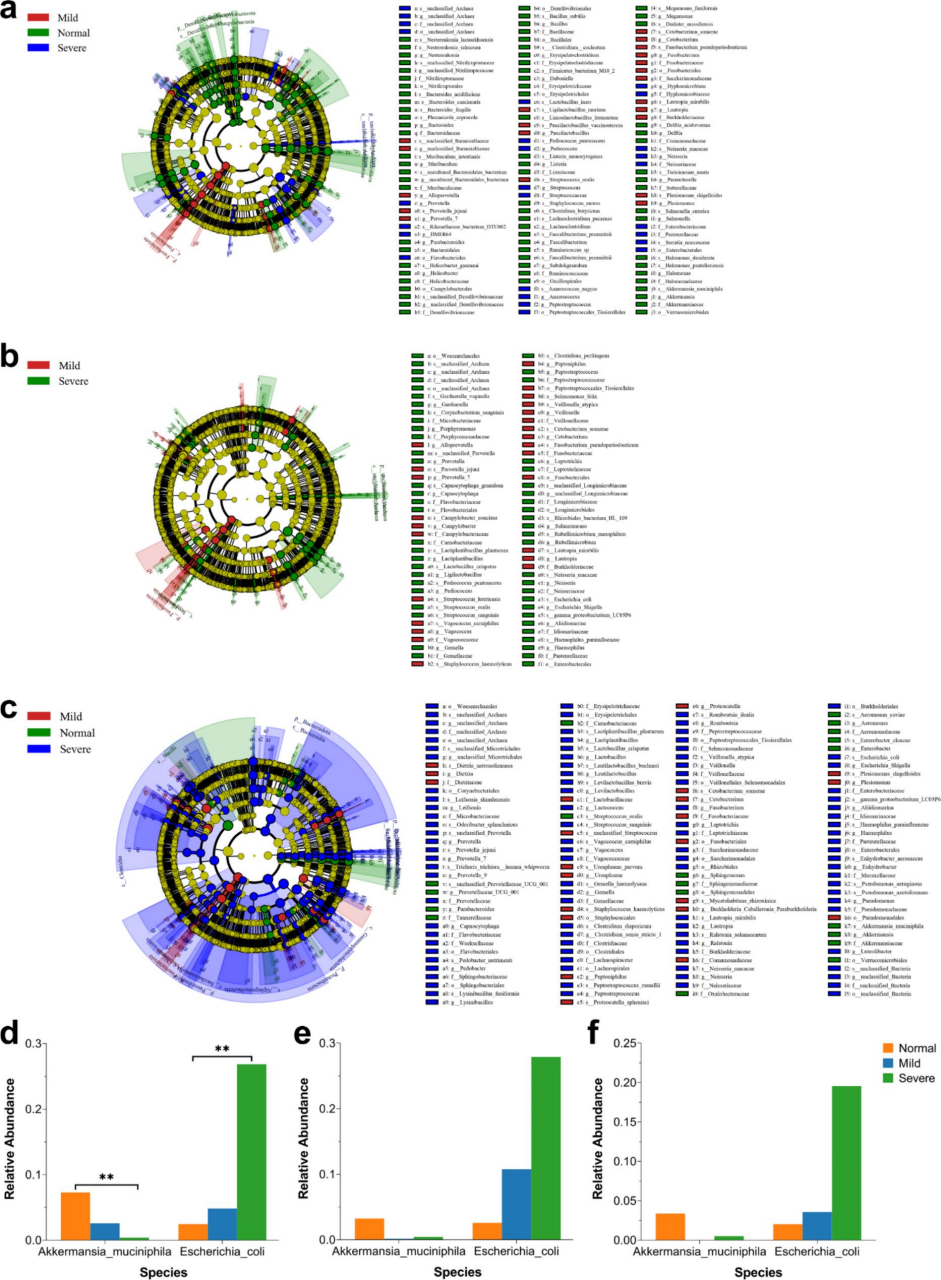
The analysis of inter-group significant differences between normal, mild, and severe groups. The differential dominant microorganisms obtained through LEfSe analysis based on (**a**) 33 samples collected on Day 1, (**b**) 33 samples collected on Day 3, and (**c**) 35 samples collected on Day 5. Differences in the relative abundance of species at the species level obtained through ANOVA analysis based on (**d**) 33 samples collected on Day 1, (**e**) 33 samples collected on Day 3, and (**f**) 35 samples collected on Day 5

In terms of the relative abundance of gut microbiota, the species composition of the normal, mild, and severe IVH group at the phylum, genus, and species levels are shown in Fig. 2, and the species composition of all 101 samples at the phylum, genus, and species levels are shown in Supplementary Fig. 2. The *Firmicutes*, *Proteobacteria*, and *Bacteroidota* were common major three phyla, and *Enterococcus*, *Escherichia Shigella*, and *Achromobacter* were common major three genera in all groups. At the species level, comparing the community composition and structure of gut microbiota in preterm infants with different IVH severity levels (LDA > 3), we found that there were differences in *E. coli* and *A. muciniphila* among the three groups (shown in Fig. 3). Among them, as the severity of IVH deepened, the relative abundance of *E. coli* continued to increase, and the relative abundance of *A. muciniphila* in preterm infants with IVH is lower than that in normal premature infants (shown in Fig. 3. d-f).

**Fig. 4:**
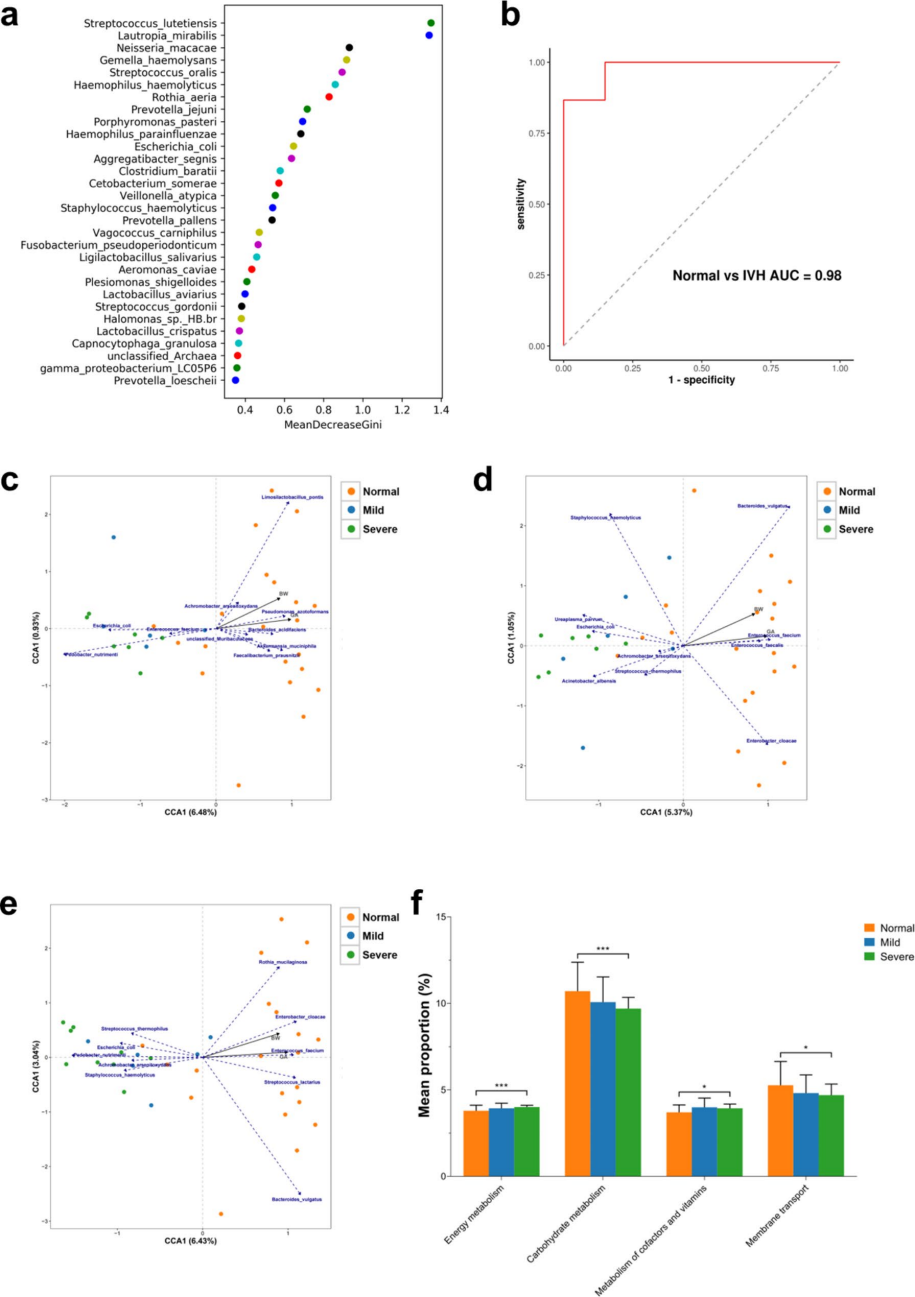
The association analysis, together with functional gene prediction analysis. (**a**) The random forest analysis at the species level. (**b**) ROC curves and their corresponding AUCs employing 7 species. The RDA/CCA analysis of GA and BW environmental factors at the species level on (**c**) Day 1, (**d**) Day 3, and (**e**) Day 5. (**f**) The functional difference diagram of PICRUSt2 function prediction based on KEGG

We conducted random forest analysis on the gut microbiota at the species level based on all 101 samples, to identify key species that have a significant impact on the differences in gut microbiota between normal preterm infants and preterm infants with IVH. We found that *S. lutetiensis*, *L. mirabilis*, *N. macacae*, *G. haemolysans*, and *S. oralis* were important in distinguishing between the two groups (shown in Fig. 4. a). Therefore, we constructed the best classifier model using the above five bacteria and the 2 major diverse bacteria (*E. coli* and *A. muciniphila*) to conduct a leave-one-out cross-validation (LOOCV). The area under curve (AUC) analysis results indicate that the above seven bacteria can effectively distinguish IVH premature infants from normal preterm infants (shown in Fig. 4. b).

#### Association analysis and functional gene prediction analysis

We also analyzed the association of gut microbiota among the normal, mild, and severe IVH groups (shown in Fig. 4. c-e). We found a certain correlation between these two environmental factors, BW and GA, and the gut microbiota of premature infants on Day 1, Day 3, and Day 5. By predicting the functional abundance of gut microbiota in premature infants with different IVH severity levels, we found significant differences in energy metabolism, carbohydrate metabolism, metabolism of cofactors and vitamins, and membrane transport between the normal and severe IVH groups (shown in Fig. 4. f). Among them, the mean proportion of carbohydrate metabolism and membrane transport in the normal group is higher than that in the severe IVH group, while the mean proportion of energy metabolism and metabolism of cofactors and vitamins is lower than that in the severe IVH group.

## Discussion

As the most common brain injury in premature infants, IVH currently lacks effective methods for screening and identifying high-risk populations before injury occurs, and the relationship between IVH and cognitive impairment mechanisms is still unclear, lacking effective methods for early intervention to improve prognosis. Recently, studies have shown a close relationship between gut microbiota and brain development and cognitive function [37]. The imbalance of gut microbiota in the early postnatal period may disrupt brain development through the “gut-brain axis”, leading to brain injury [38]. However, there is little research on whether the gut microbiota is involved in the occurrence of IVH, and the relationship between IVH and gut microbiota. This study utilized high-throughput sequencing technology to investigate the characteristics of gut microbiota in the early postnatal period and the relationship between IVH and gut microbiota in premature infants with different IVH severity levels. It was found that there was a close relationship between the two, and a significant difference in gut microbiota among premature infants with different IVH severity levels. The results of this study are of great significance for exploring new clinical risk assessment biomarkers, exploring new safe and effective therapeutic targets, and improving the cognitive and developmental prognosis of premature infants with IVH.

In this study, we found that the gut microbiota of premature infants in the early postnatal period is closely associated with the IVH status, and the presence or absence of IVH has the greatest impact on the gut microbiota. We found that there were differences in the gut microbiota of premature infants with different IVH severity within Day 5 in terms of alpha diversity, beta diversity, differential dominant microbiota, and relative abundance. Among them, compared to normal premature infants, the alpha diversity in the premature infants with IVH was significantly increased, but no differences in alpha diversity were found between mild and severe IVH premature infants. In addition, we also found that as age increases, the differences in gut microbiota of premature infants with different degrees of IVH continue to increase, and the trend of changes with severity of IVH becomes more and more obvious.

Previous studies have suggested that early fecal samples from premature infants are dominated by *Firmicutes*, gradually transitioning to *Proteobacteria*, while the relative abundance of *Enterobacteriaceae*, including *Klebsiella* and *Escherichia*, is higher than that of *Bifidobacterium* [39]. The results of this study are consistent with the previous reports.

The differential analysis of different IVH groups in this study using LEfSe revealed that *E. coli* and *A. muciniphila* are the main differential bacteria between the IVH and normal groups. Combining the random forest model to predict different IVH, it was found that there was a good ROC curve. Therefore, we can use these bacteria to accurately predict and identify potential IVH infants. Previous studies have found that *E. coli* can produce short-chain fatty acids and metabolites such as lactic acid and acetic acid [40]. *E. coli* has an inhibitory effect on intestinal inflammation and can also assist the host in producing nitrates, inhibiting the growth of *Pseudomonas aeruginosa* [41, 42]. *Acinetobacter albensis* was first identified from water and soil and has not been reported in the gut microbiota. However, in the study of the gut microbiota in patients with multiple sclerosis, it was found that *Acinetobacter calcoaceticus*, also belonging to the *Acinetobacter* genus, has a significant increase in relative abundance in the gut microbiota of patients with multiple sclerosis. The genus *Akkermansia* has been shown to appear in the first year of life and to gradually increase in abundance until adulthood [43]. Caloric restriction or starvation increases the abundance of *A. muciniphila* in the human and animal gut. *A. muciniphila* has proven efficacy to improve obesity, type 2 and type 1 diabetes mellitus, hepatic steatosis, intestinal inflammation and different cancers (colon cancer, response to immune checkpoints) in mice. In vitro, protein 9 (P9) activate the enteroendocrine L cells and stimulates glucagon-like peptide-1 (GLP1) and regulate inflammation, fatty acid oxidation and glucose metabolism [44]. Interestingly, the functional abundance of gut microbiota showed significant difference in energy metabolism, carbohydrate metabolism, membrane transport, and the metabolism of cofactors and vitamins. These differences suggest that the gut microbiota of preterm infants with severe IVH may have altered metabolic capabilities, potentially affecting the overall energy balance and nutrient absorption. Alterations in carbohydrate metabolism could impact the availability of essential nutrients required for growth and development, while changes in membrane transport and cofactor metabolism could influence the gut’s ability to maintain homeostasis and respond to environmental challenges.

Future study focusing on stool metabolome could help reveal the key molecular that involved in related metabolic pathways. Experimental models, including germ-free and gnotobiotic animal models, could be employed to dissect the underlying biological mechanisms. Integrating multi-omics approaches, including genomics, transcriptomics, proteomics, and metabolomics, will offer a holistic view of the host-microbiota interactions. This could lead to the identification of novel biomarkers for early diagnosis and personalized therapeutic strategies. It is expected to provide important insights for further exploring potential new methods based on gut microbiota and its metabolites for identifying high-risk populations for possible brain injury, exploring new safe and effective therapeutic targets, and improving the cognitive and developmental prognosis of premature infants with IVH.

However, there were also some limitations of our study. This study is currently a single-center study, and there may be differences in gut microbiota in the early postnatal period of premature infants from other hospitals, including those with IVH, which needs to be validated through multicenter studies. In addition, this study only explored the characteristics and differences of gut microbiota in premature infants with different degrees of IVH, and has not yet conducted research on the relationship between long-term prognosis and gut microbiota characteristics. In future research, follow-up can be conducted for this cohort, and it is expected to discover a correlation analysis between early gut microbiota and IVH prognosis.

## Conclusions

The gut microbiota in the early postnatal period of premature infants is closely associated with the IVH status. As age increases, the differences in gut microbiota of premature infants with different degrees of IVH continue to increase, and the trend of changes with severity of IVH becomes more and more obvious. *E. coli*, *A. muciniphila*,* S. lutetiensis*,* L. mirabilis*,* N. macacae*,* G. haemolysans*,* and S. oralis* can effectively distinguish between IVH infants and normal premature infants. The results indicate that gut microbiota is expected to provide effective therapeutic targets for the diagnosis and treatment of IVH.

## Electronic supplementary material

Below is the link to the electronic supplementary material.

Supplementary Material 1

## Data Availability

The datasets (Clean-CCS) analyzed during the current study are available in the NCBI repository, http://www.ncbi.nlm.nih.gov/bioproject/1086214.

## Abbreviations

ADHD: Attention deficit hyperactivity disorder
ANOSIM: Analysis of similarities
ANOVA: Analysis of variance
AUC: Area under curve
BDNF: Brain-derived neurotrophic factor
CCS: Circular consensus sequencing
Day: Postnatal day
DOL: Day of life
GA: Gestational age
GLP1: Glucagon-like peptide-1
IVH: Intraventricular hemorrhage
KEGG: Kyoto encyclopedia of genes and genomes
LefSe: Linear discriminant analysis effect size
LOOCV: Leave-one-out cross-validation
MEBM: Maternally expressed breast milk
OTU: Operational taxonomic unit
PCoA: Principal co-ordinates analysis
PCR: Polymerase chain reaction
PDA: Patent ductus arteriosus
PICRUSt2: Phylogenetic investigation of communities by reconstruction of unobserved states2
RDA/CCA analysis: Redundancy analysis / canonical correspondence analysis
