# Supplementary material for: Characteristics of gut microbiota of premature infants in the early postnatal period and their relationship with intraventricular hemorrhage
Source: BMC Microbiol. 2024 Dec 2;24:513. doi: 10.1186/s12866-024-03675-w (PMC11610090; doi:10.1186/s12866-024-03675-w)
Supplement: Supplementary file 1 — Supplementary Material 1 [file 12866_2024_3675_MOESM1_ESM.docx]

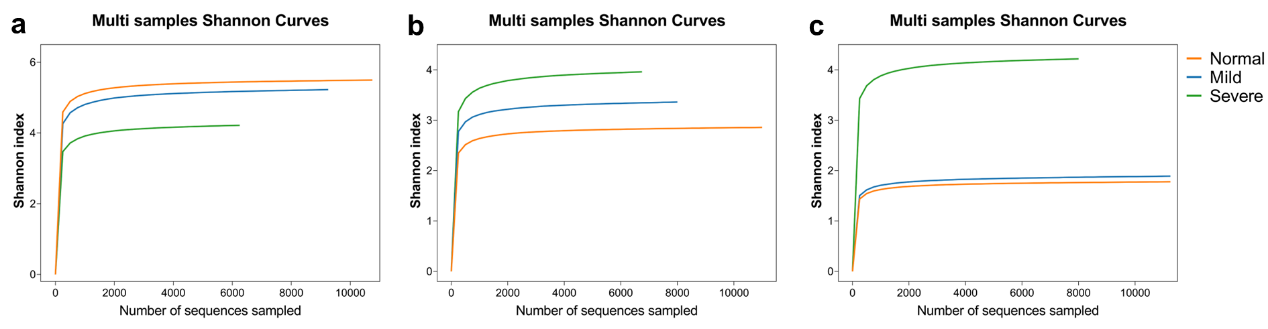


**Supplementary Fig. 1. The Rarefaction curves based on Shannon index between normal, mild, and severe groups.** The Rarefaction curves based on Shannon index between normal, mild, and severe groups based on (a) 33 samples collected on Day 1, (b) 33 samples collected on Day 3, and (c) 35 samples collected on Day 5.


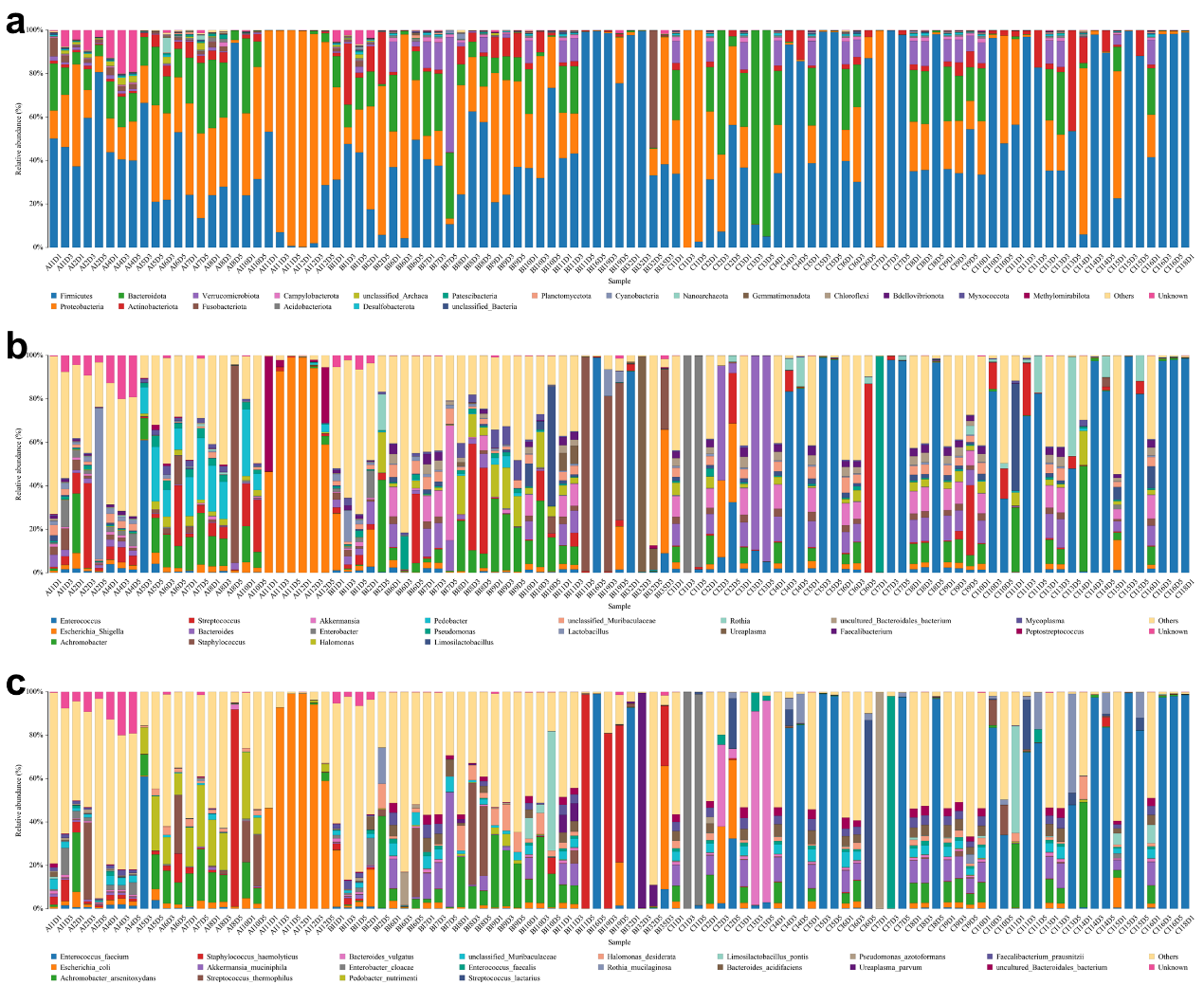


**Supplementary Fig. 2.** The analysis of species distribution differences between all 101 samples. The species distribution bar charts at the (a) phylum, (b) genus, and (c) species level.

**Supplementary Table 1: Clinical characteristics of gestational age distribution in enrolled premature infants.**

| **Descriptive variable** | **Day 1 group**  **n=33** | **Day 3 group**  **n=33** | **Day 5 group**  **n=35** |
| --- | --- | --- | --- |
| **Normal, n (%)** | 21(63.7) | 20(60.1) | 20(57.1) |
| **Mild IVH, n (%)** | 5(15.1) | 6(18.2) | 6 (17.1) |
| **Severe IVH, n (%)** | 7(21.2) | 7 (21.2) | 9 (25.8) |

*IVH: intraventricular hemorrhage; Day, postnatal day.*
